# Supplementary material for: ER shaping proteins guide spindle elongation and division during rapid cleavage mitoses
Source: bioRxiv. 2026 Jul 16:2026.07.15.738729. Preprint. [Version 1] doi: 10.64898/2026.07.15.738729 (PMC13405190; doi:10.64898/2026.07.15.738729)
Supplement: Supplementary file 1 [file NIHPP2026.07.15.738729v1-supplement-1.pdf]

bioRxiv preprint doi: https://doi.org/10.64898/2026.07.15.738729; this version posted July 16, 2026. The copyright holder for this preprint (which was not certified by peer review) is the author/funder, who has granted bioRxiv a license to display the preprint in perpetuity. It is made available under a CC-BY 4.0 International license.

Supplemental Figure 1

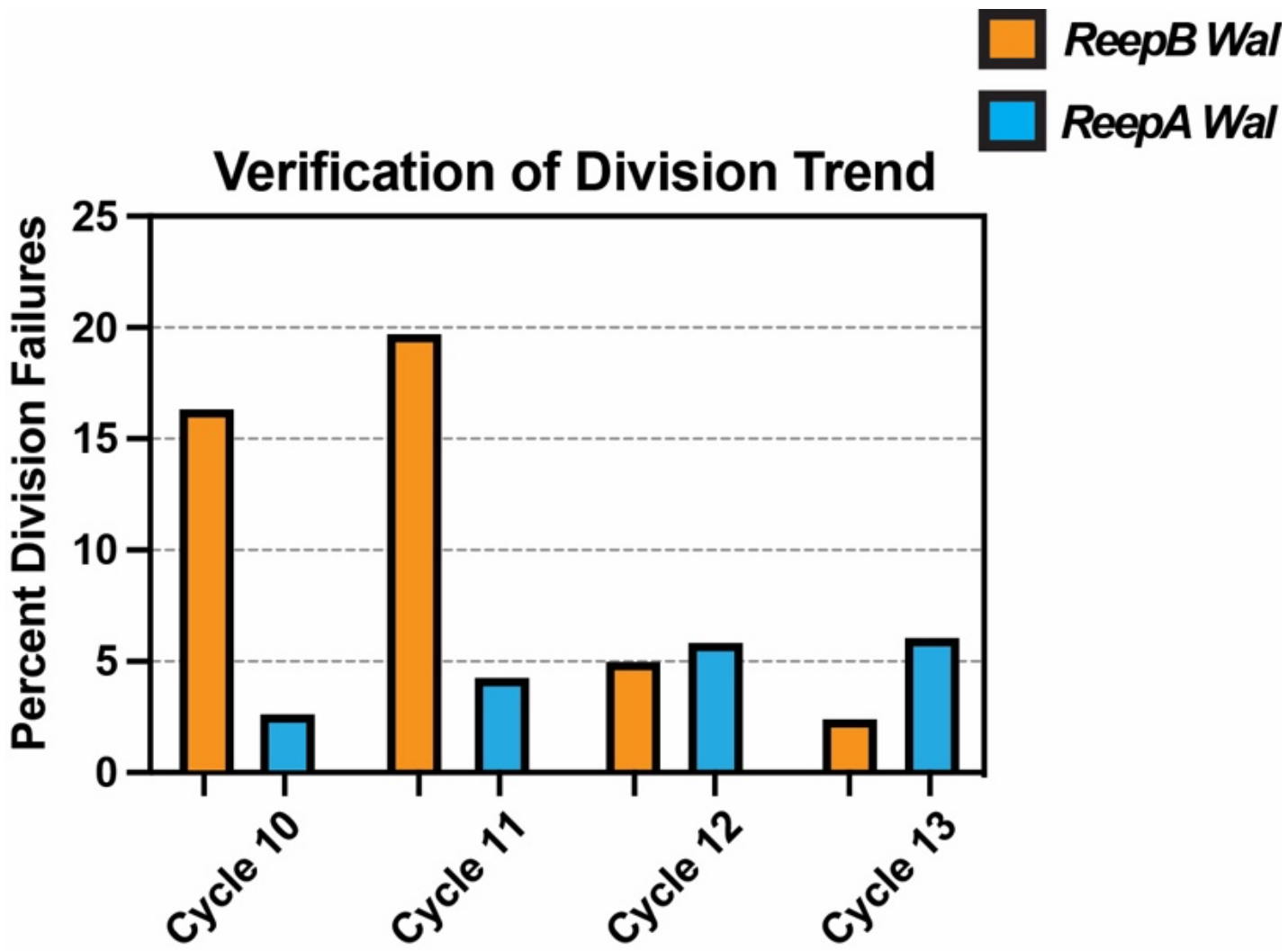

Supplemental Figure 1: Independent shRNA lines demonstrate similar defects in mitoses during syncytial cycle. shRNA lines against ReepA and ReepB created in pWalium22 with unique targeting sequences from constructs reported in Figure 1.
